# Electronic Technologies: Can They Alter the Human Aspect of Medical Care?

**DOI:** 10.6004/jadpro.2012.3.2.1

**Published:** 2012-03-01

**Authors:** Pamela Hallquist Viale

The use of electronic health-care records (EHRs) and communication technologies has expanded considerably in the past 5 years. Electronic health-care records and computerized physician order entry have contributed to greater accuracy in care and reduced medical errors in the high-risk oncology setting (Harshberger et al., 2011). And although the use of EHRs requires training as well as ongoing vigilance and assessment, studies have documented staff satisfaction with the ability of EHRs to provide current and comprehensive patient information (de Veer & Francke, 2010; Harshberger et al., 2011).

## Benefits

In addition to EHRs, many health-care providers also use wireless personal electronic devices to access immediate information on patient data, get updated drug information, and input patient information into the EHR. These devices include, but are not limited to, laptops, tablets, hand-held computers, and smartphones. The devices provide the ability to communicate via longer text messages; they also allow the user to access applications that give drug information, enable medical calculations, and even provide updated evidence-based guidelines (Moore, 2011). For health-care providers who previously depended on hard copy reference guidelines and medical texts for current information, personal electronic devices are a welcome addition to patient care. These electronic advances are a major focus of our *Tools & Technology* feature in the *Journal of the Advanced Practitioner in Oncology*. As a nurse practitioner working with patients in oncology, I can say that the improvements in electronic health-care technology have made a significant difference; as a consumer of medical care, though, these "improvements" may have a downside.

## Potential Limitations

A recent *New York Times* article pointed out that the increased use of wireless personal electronic devices creates the potential for distraction (Richtel, 2011). Although computers, smartphones, and other devices provide current data to assist in delivering care, providers can become too focused on the device screen instead of the patient (Richtel, 2011). The problem has been termed "distracted doctoring," referring to providers who are so attached to their phones, computers, and tablets that they may miss valuable patient information (Richtel, 2011). Dr. Peter Papadakos, an anesthesiologist and director of critical care at the University of Rochester Medical Center, noted recently in an editorial that the previous method of communication and patient care has changed to a new model with electronic records and downloading of patient care data. He also notes that today’s health-care workers rarely receive information directly from their patients; instead they access the health history on a computer (Papadakos, 2011). As Dr. Papadakos points out, providers and other health-care workers may also be accessing games, using non–work-related applications, or answering personal emails, distracting them from patient care.

I recently experienced this phenomenon during an annual visit to my health-care provider. Although the physician nodded his head appropriately, he was glued to the computer screen while he input pertinent data from my oral history. The physician may have glanced at my actual face twice during a 10-minute discussion, after which a brief, 2-minute physical exam followed. He pulled out a handheld device to check a possible drug-drug interaction and transcribed the data into the EHR. My overall feeling was mixed; as a fellow provider I sympathized with his difficulties trying to input the data into the correct spots in the EHR, but as a patient I felt a distinct lack of personal care.

## Our Practice Patterns

There is no doubt that the evolution of electronic medical records has improved care; the jury is still out on the use of personal electronic devices. There is little published information on the effectiveness of these devices in health care. A 2008 study examined the value of wireless personal digital assistants (PDAs) for practice by questioning a group of advanced practice nurses. Although some of the nurses were reluctant to integrate the new technologies into practice because of cost and the short technological life of the devices, most reported the pervasiveness of the new technology and its acceptance by advanced practice nurses (Garrett & Klein, 2008). Stroud and colleagues (2009) described the prevalence and patterns of PDA use by 126 nurse practitioners in 2009; 64% of the participants used the devices, with drug reference software the most useful and frequently installed application. This number is undoubtedly higher today.

## Conclusion

While I applaud electronic devices that help to improve patient care in the medical setting, I think caution is warranted. The value of face-to-face contact with your patient is immeasurable; health-care providers should take care to respect the patient encounter while integrating electronic aids to improve care. As electronic technologies continue to advance at a rapid pace, advanced practitioners will continue to use them in practice and benefit from the information they can quickly provide. But the patient will always come first.
